# Microenvironmental Conditions Drive the Differential Cyanobacterial Community Composition of Biocrusts from the Sahara Desert

**DOI:** 10.3390/microorganisms9030487

**Published:** 2021-02-25

**Authors:** Smail Mehda, Maria Ángeles Muñoz-Martín, Mabrouka Oustani, Baelhadj Hamdi-Aïssa, Elvira Perona, Pilar Mateo

**Affiliations:** 1 Departamento de Biología, Facultad de Ciencias, Universidad Autónoma de Madrid, 28049 Madrid, Spain; mehda-smail@univ-eloued.dz (S.M.); mangeles.munnoz@uam.es (M.Á.M.-M.); elvira.perona@uam.es (E.P.); 2 Laboratory of Biogeochemistry of Desert Areas, University of Ouargla, 30000 Ouargla, Algeria; hamdi_30@yahoo.fr; 3 Department of Agronomy, Faculty of Life and Natural Sciences, University of El Oued, 39000 El Oued, Algeria; 4 Laboratory of Saharan Bio-Resources: Preservation and Development, University of Ouargla, 30000 Ouargla, Algeria; belsam.oustani@yahoo.fr

**Keywords:** biocrust, cyanobacteria, Sahara Desert, polyextreme conditions, hyperarid deserts

## Abstract

The Sahara Desert is characterized by extreme environmental conditions, which are a unique challenge for life. Cyanobacteria are key players in the colonization of bare soils and form assemblages with other microorganisms in the top millimetres, establishing biological soil crusts (biocrusts) that cover most soil surfaces in deserts, which have important roles in the functioning of drylands. However, knowledge of biocrusts from these extreme environments is limited. Therefore, to study cyanobacterial community composition in biocrusts from the Sahara Desert, we utilized a combination of methodologies in which taxonomic assignation, for next-generation sequencing of soil samples, was based on phylogenetic analysis (16S rRNA gene) in parallel with morphological identification of cyanobacteria in natural samples and isolates from certain locations. Two close locations that differed in microenvironmental conditions were analysed. One was a dry salt lake (a “chott”), and the other was an extension of sandy, slightly saline soil. Differences in cyanobacterial composition between the sites were found, with a clear dominance of *Microcoleus* spp. in the less saline site, while the chott presented a high abundance of heterocystous cyanobacteria as well as the filamentous non-heterocystous *Pseudophormidium* sp. and the unicellular cf. *Acaryochloris*. The cyanobacteria found in our study area, such as *Microcoleus steenstrupii, Microcoleus vaginatus, Scytonema hyalinum, Tolypothrix distorta,* and *Calothrix* sp., are also widely distributed in other geographic locations around the world, where the conditions are less severe. Our results, therefore, indicated that some cyanobacteria can cope with polyextreme conditions, as confirmed by bioassays, and can be considered extremotolerant, being able to live in a wide range of conditions.

## 1. Introduction

The Sahara is the largest desert in the world, is subjected to a wide range of climatic conditions and is one of the most hyperarid regions on Earth, with an aridity index <0.05 [1]. This index reflects the water deficit in these systems by expressing the ratio of precipitation to potential evapotranspiration, and arid regions are those with an arity index below 1 [2].

Cyanobacteria are considered pioneers in terrestrial ecosystems, since they can cope with drought [3], high temperature [4] and prolonged UV radiation [5]. The colonization of desert soils by cyanobacteria leads to soil aggregation and erosion protection, carbon and nitrogen fixation, an increase in soil organic matter, and facilitation of the subsequent growth of lichens, mosses, and finally vascular plants [6,7].

Natural succession starts with the colonization of bare soils by filamentous cyanobacteria, which form microbiotic assemblages with heterotrophic bacteria, archaea, algae and fungi living in the top millimetres, establishing biological soil crusts (biocrusts), which cover most soil surfaces in deserts and can cover up to 70% of the total area [1]. As key players in biocrust development, cyanobacteria have received much attention for the restoration of soils in drylands ([8] and references therein). For instance, selected cyanobacterial strains grown in liquid culture have been inoculated over large hyperarid areas in China to restore soils degraded by intensifying desertification [9,10]

Soil degradation and the subsequent world desertification process are major environmental problems. Awareness that soil is one of the most important and most vulnerable resources and that land is degrading in hyperarid areas because of various factors, including climatic variations and human activities, is growing. Drylands are the most sensitive areas for desertification worldwide [11], and African lands in the southern Mediterranean basin are more affected by desertification than other lands around the world [12].

To address the global problem of soil desertification, identifying and monitoring biota should be the first steps, and if degraded soils are to be restored and/or the spread of desertification is to be reversed using cyanobacteria-based approaches, a deep knowledge of the native and dominant cyanobacteria of these ecosystems is necessary. However, cyanobacterial biocrust communities remain unknown or poorly characterized in many regions, especially in Africa, where studies have been carried out, principally in South Africa [13,14,15], with North Africa and West Asia, which have the lowest coverage across soil macroecological studies [16].

In addition, the Sahara Desert in Algeria harbours typical endorheic systems that consist of saline lake ecosystems, locally called chotts and sebkhas, with the typical alternation of a drought phase in summer and flooding in winter, which are even less studied. In these areas, the surface water table in the lower parts of the landscape (playa) often evaporates to dryness, inducing very intense salinization, resulting in the formation of brines and surfaces, often encrusted with several centimeters of thick salt crust [17,18, 19].

Thus, the aim of the present study was to use 16S rRNA Illumina MiSeq sequencing to analyse and compare the diversity and abundance of cyanobacterial communities of two saline localities, separated by approximately 40 km but clearly differing in their characteristics, in this zone of the Sahara Desert. One of the locations was a dry salt lake (a chott), but the other was an extension of sandy, slightly saline soil characterized by the presence of typical well-developed cyanobacterial biocrusts.

In recent years, there has been an increase in the application of next-generation sequencing (NGS) technologies in environmental microbiology studies, of which one of the most widely used is the Illumina sequencing platform. However, taxonomic affiliations in most studies are based only on bioinformatics tools, which rely on the sequences present in databases, which currently contain a relatively low number of cyanobacterial sequences. Consequently, assignments are often possible at only the family or even order level, since no matches are available in the databases. Thus, the challenge of linking characterized genetic entities (operational taxonomic units) with identified biocrust organisms is an essential step in NGS analysis [20,21].

Therefore, in our survey, as previously carried out in previous studies [22,23,24], we first isolated cyanobacterial strains from the analysed soils, which were morphologically and genetically (16S rRNA) characterized. We also analysed original soil field samples by microscopy and compared them with morphotypes found in the cultures, together with their genetic characterization, which allowed us to obtain better taxonomic assignments in the NGS analysis. In addition, given the harsh conditions of hyperarid ecosystems, such as the studied habitat, cyanobacteria inhabiting these ecosystems might have developed strategies to resist high temperatures and dry conditions; therefore, a series of thermophysiological and desiccation bioassays were carried out to explain differences in the relative abundance of the cyanobacteria at the sites and to relate these findings to the global distribution of these cyanobacteria. This integrative approach allowed us to compare the cyanobacterial diversity from two close but different hyperarid environments and to advance our understanding of the influence of the heterogeneity of microenvironments on the structure and composition of cyanobacterial communities.

## 2. Materials and Methods

### 2.1. Study Area and Site Descriptions

**Figure 1:**
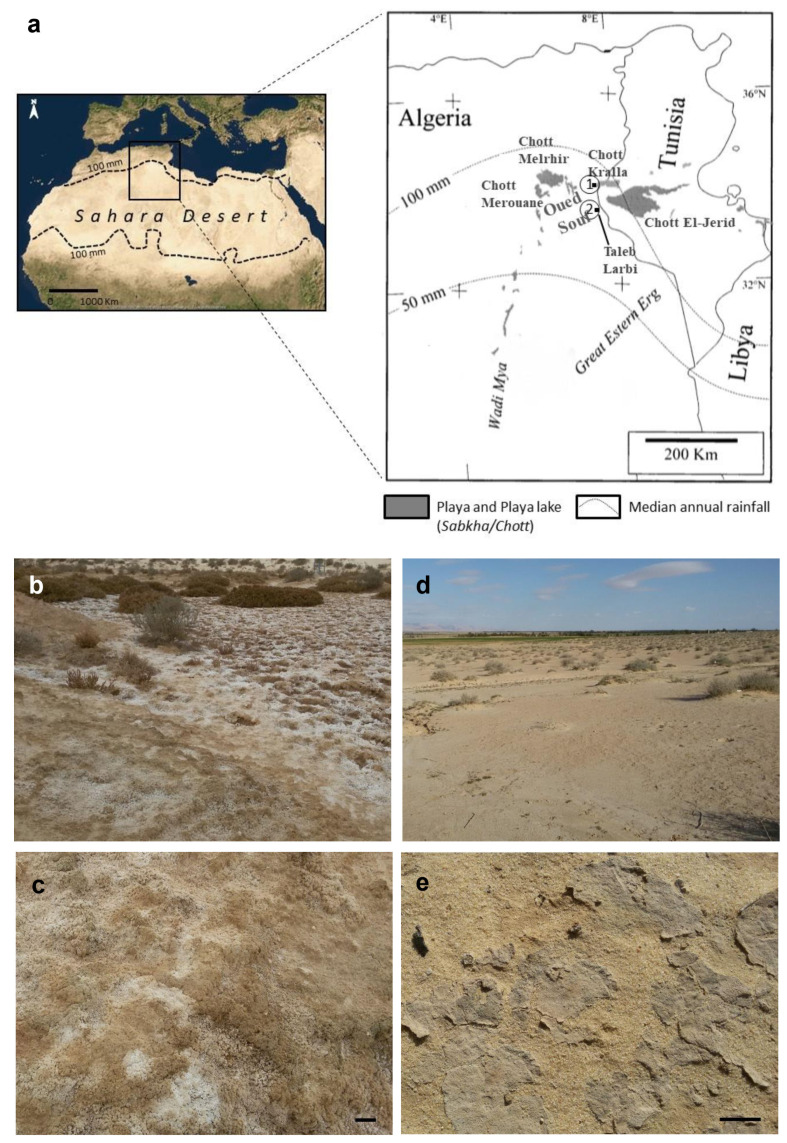
(**a**) Location of the studied area and sampling sites. 1. Chott Kralla, 2. Taleb Larbi. (**b**) General view of Chott Kralla. (**c**) Biocrust from Chott Kralla. (**d**) General view of Taleb Larbi. (**e**) Biocrust from Taleb Larbi. Scale bar 2 cm.

This study was conducted in the Oued Souf area of the northern Sahara Desert (Algeria) (Figure 1a). The area is characterized by a dry climate that lasts throughout the year. It is of the hyperarid Mediterranean type with a temperate winter, and a permanent drought. It is particularly contrasting despite the relatively northern latitude [25]. The mean annual rainfall is 67.37 mm (2009–2018). The average annual temperature is 24.79 °C, with an average maximum of 46.26 °C in July and an average minimum of 7.23 °C in January. Winds dominate, with an average speed of 4.18 m/s. The evaporation is very intense, with a yearly total of 2463.81 mm. The annual mean relative humidity is 45.16%, although winters are relatively humid, reaching a value of 63.82% in December [26].

Biocrust samples were collected at two sites located in the study area. Site 1 (34°04′08.6″ N, 7°27′53.9″ E) is located inside Chott Kralla (Figure 1a–c), a shallow water body that evaporates to dryness, leaving mudflats or playas, whose soil is characterized by the presence of a whitish salt layer on the surface (3 mm) and is classified, according to IUSS-Working-Group-WRB, (2015) [27], as Calcic Gypsic Solonchak (Hypersalic, Aridic). The biocrusts from this site are hardly detectable because of the presence of distinctive salt crusts from the chott (Figure 1b,c). Due to the nature of the site, halophytic plants colonize Chott Kralla (*Limoniastrum guyonianum* Dur., *Tamarix boveana* Bunge., *Zygophyllum album* L., *Sueda mollis* (Desf.) Del.). Site 2 (33°43′04.3″ N, 7°26′28.2″ E) is located near the Taleb Larbi locality. It is an extension of sandy soil characterized by the presence of typical cyanobacterial biocrusts (Figure 1d,e). The soil is classified as Calcic Gypsic (Arenic, Aridic) [27], and relatively different vegetation types cover the Taleb Larbi site (*Matricaria pubescens* (Desf.) Schultz., *Calligonum comosum* L’her., *Diplotaxis harra* (Forsk.) Boiss., *Cornulaca monacantha* Del., *Malcolmia aegyptiaca* Spr., *Retama retam* Webb.).

### 2.2. Biocrust Sampling

The biocrust samples were collected from the two aforementioned sites in December 2018, following a previously described procedure [23]. At each site, an area of 25–50 m^2^ was inspected to choose places with biocrusts, and at least three biocrust samples were collected at each site. The samples were allowed to dry completely, sealed in zippered plastic bags, and stored in the dark at room temperature until use. A preliminary determination of the major cyanobacterial morphotypes and their relative abundances was carried out by direct microscopy of wet samples. Then, representative subsamples, according to microscopic inspection and of equal size, were selected, mixed together and homogenized with a mortar and pestle to form a composite sample. These multiple samples integrated the field patchiness of the communities [22,23,24].

### 2.3. Characterization of Physicochemical Soil Properties

The soil samples were air-dried, passed over a 2 mm sieve and ground using a mortar and pestle before physicochemical analyses. The pH was measured at a substrate:solution ratio of 1:2.5 in distilled water with a glass electrode [28]. The electrical conductivity (EC) was measured in a soil and distilled water suspension of 1:5 [29]. The total carbon content was determined by sulfochromic oxidation [30], followed by titration of the excess K_2_Cr_2_O_7_ with FeSO_4_(NH_4_)_2_SO_4_·6H_2_O. Kjeldahl’s method was adopted to determine the total dosage of nitrogen [31]. Finally, the gypsum content in the samples was measured by analysing the total sulphates as barium sulphate using the gravimetric method based on the precipitation of sulphate ions in acidic media [32]. Bernard’s calcimeter method was used to determine the total dosage of limestone [33].

### 2.4. Strain Isolation and Culture Conditions

Strain isolation was accomplished by two methods. In the first one, the composite samples were ground with a mortar, and 0.2 g was mixed with 0.5 mL of BG11_0_ culture medium [34], incubated for 30 min at room temperature and then centrifuged (3000× *g*, 30 s). Two aliquots of 0.2 mL of the supernatant were separately cultured on agar plates (30 mL, 1% agar) with BG11_0_ or BG11 culture media containing cycloheximide (0.1 mg·mL^−1^) to avoid fungal contamination. To isolate strains from the Chott Kralla site, 0.2 mL of soil extract was autoclaved and added to the agar plates, as the first isolation attempts were unsuccessful. The soil extract was prepared by mixing 2 g of the soil sample and 18 mL of BG11_0_ medium for half an hour and centrifuging for 2 min at 5000× *g*. The plates were incubated in a growth chamber at 28 °C and 20–50 mmol photon m^−2^·s^−1^ and allowed to grow for approximately 4 weeks, as previously described [35,36]. The procedure was replicated to isolate a large number of cyanobacterial strains, with a total of 11 agar plates for samples from site 1, and 17 agar plates for samples from site 2 (approximately half and half of each culture medium). Each strain was isolated from the colonies by selecting single trichomes using pulled capillary pipettes or forceps under a dissecting microscope (Leica, Leica Microsystems, Wetzler, Germany). The isolated strains were transferred to multiwell plates with liquid BG11_0_ (for heterocystous cyanobacteria) or BG11 (for non-heterocystous cyanobacteria) and maintained at 28 °C and 20–50 mmol photon m^−2^·s^−1^ [22]. To isolate strains from endolithic and hypolithic samples, for comparison with biocrusts samples, some variations in the procedure were carried out. For the endolithic samples, a preliminary inspection of the colonized areas in the stone was carried out by direct microscopy. Then, representative areas were selected, scraped from the stone, mixed, and homogenized with a mortar and pestle to form a composite sample, which was cultured on the agar plates as indicated above. Regarding the hypolithic samples, the bottom of the stones was scraped with a small knife and brushed, and then mixed for a composite sample and cultured, as carried out for endolithic samples.

For the second isolation method, bundles of filaments were manually isolated by micromanipulation of the biocrust samples after reactivation by the addition of distilled water under a dissecting microscope with watchmaker forceps, as previously described [22,35]. Nine strains were isolated by this procedure. The isolated bundles or filaments were further separated and cleaned by dragging them over a solid agarose medium, observed under a compound microscope to confirm the presence of only one morphotype, and subsequently inoculated in multiwell plates with liquid BG11. Cycloheximide (0.1 mg·mL^−1^) was also added to avoid fungal contamination. After further incubation, cultures obtained by the two methods were transferred to flasks with liquid culture medium and grown under the same laboratory conditions. Cultures were named after the site where the strain was isolated followed by a number, SBC (saline biocrusts) from Chott Kralla and LSB (less saline biocrusts), from Taleb Larbi, and included in the culture collection of the Universidad Autónoma de Madrid (UAM).

### 2.5. Morphological Characterization

Morphological characterization included the occurrence of specialized cells, such as heterocysts or akinetes, as well as the presence of sheaths, trichome characteristics or cell and colony morphology. This characterization was carried out using an Olympus BH2-RFCA photomicroscope (Olympus, Tokyo, Japan). The cyanobacterial characteristics of the natural samples as well as the cultures were compared with the information provided in the taxonomic keys of Komárek (2013) and Komárek and Anagnostidis (1999, 2005) [37,38,39].

### 2.6. DNA Isolation and Amplification of the 16S rRNA Gene of the Isolated Strains

Total genomic DNA was extracted from the isolated cultures with an UltraClean^®^ Microbial DNA Isolation Kit (MO BIO Laboratories, Inc., Carlsbad, CA, USA) with a previously described modification [22,40] to break the exopolysaccharides surrounding many of the cyanobacterial cells. This involved a three-cycle step that consisted of freezing 0.3 mL aliquots of cyanobacterial suspensions of each culture in liquid nitrogen, breaking them down with an adapted drill, and melting them in a 60 °C water bath.

The 16S rRNA gene was amplified by PCR using the primer 27F (5′-AGAGTTTGATCCTGGCTCAG-3′) as the forward primer [41] and the primer B23SR (5′-CTTCGCCTCTGTGTGCCTAGGT-3′) as the reverse primer [42] under conditions previously described by [43]. This reaction produced amplification fragments of approximately 2000 bp that spanned the 16S rRNA gene and the intergenic region between the 16S and 23S rRNA genes. PCR products were cloned into pGEMR-T Easy Vector Systems (Promega, Madison, WI, USA) and transformed into *Escherichia coli* DH5α. Positive clones were verified by PCR using the universal primers T7 (5′-TAATACGACTCACTATAGGG-3′) and SP6 (5′-ATTTAGGTGACACTATAG-3′). Plasmid DNA from positive clones (1 or 2 for each strain) was extracted using a Wizard Miniprep kit (Promega, Madison, WI, USA) and commercially sequenced by the Parque Científico de Madrid, Unidad de Genómica y Proteómica, Facultad CC Biológicas—UCM using the aforementioned T7 and SP6 primers and the primer 16S 684F (5′- GTGTAGCGGTGAAATGCGTAGA-3′). Partial sequences were aligned into contigs and were manually corrected to remove ambiguous sites using BIOEDIT (version 7.2.5) [44]. Nucleotide sequences were deposited in the GenBank database under the accession numbers MW403930-MW403970.

### 2.7. Phylogenetic Analyses of 16S rRNA Gene Sequences of Isolated Strains

The 16S rRNA gene sequences of approximately 1500 bp were aligned by ClustalW multiple alignment, together with sequences with an identity value higher than 97.5% and other representative soil cyanobacterial sequences downloaded from the NCBI database and manually corrected using BIOEDIT 7.2.5 [44]. The phylogenetic trees were computed with MEGA version 7.0.21 [45] using the neighbour-joining method [46], using the *Escherichia coli* 16S rRNA gene sequence as the outgroup. The evolutionary distances were calculated using the Tajima-Nei model [47] with a pairwise deletion of gaps and missing data. The standard error was estimated with the bootstrap phylogeny test [48], using 1000 replications. The percent similarity between sequences was determined as (1-p-distance)*100.

### 2.8. Analyses of Cyanobacterial Community Composition by Amplicon Metagenomics

Genomic soil DNA was extracted and purified as previously described [23]. The variable region V3–V4 from the 16S rRNA gene was amplified by PCR using the cyanobacterial-specific primers CYA359F and 781Ra/781Rb [49] in separate reactions, and then Illumina MiSeq sequencing was used to assess the diversity and community composition of cyanobacteria. Amplicons for each sample were processed at the Genomic Service from the Universidad Autónoma de Madrid using a MiSeq sequencer (Illumina Inc., San Diego, CA, USA) with a read length of 2 × 300 bp. At least 100 000 sequences were obtained for each amplicon. Sequence data were processed using QIIME v.1.9.0 [50], following the UPARSE pipeline [51] implemented by the software USEARCH v.8.1, as described by Muñoz-Martín et al. (2020) [23]. Operational taxonomic units (OTUs) were clustered using a similarity cut-off value of 97%, and OTU representative sequences with a relative abundance higher than 0.5% in any of the sites were assigned based on comparison with isolated culture sequences from this study, NCBI database blast results, phylogenetic trees, and Greengenes and SILVA database-based assignments, as described by Muñoz-Martín et al. (2020) [23]. Alpha diversity indices (Chao1, Good’s coverage, and observed OTUs) were calculated using QIIME. The OTU sequences were deposited in the GenBank database under accession numbers MW404155-MW404176. Raw sequencing data were deposited in the NCBI Sequence Read Archive under accession number PRJNA701160.

### 2.9. Cyanobacterial Survival Bioassays

To determine survival at high temperature and desiccation, a set of experiments was carried out as previously described [22] with some modifications. Resistance to high temperatures was tested by incubating the isolated strains at 35 and 40 °C in culture medium (BG11 for non-heterocystous and BG11_0_ for heterocystous cyanobacteria) for 25 days. To avoid a possible effect of nutrient limitation and/or premature desiccation, culture medium was added once a week. Incubations were carried out in sterile polystyrene 25-well microtiter plates (IWAKI Microplate, Tokyo, Japan) with equal amounts of inoculum from each of the strains, in duplicate, in a 16:8 h light:dark period with an irradiance of 30 mmol photon m^−2^·s^−1^. Then, the cultures were left at 35 and 40 °C until total desiccation and maintained in these conditions for two months. The resilience (the ability of the cyanobacteria to recover following the previous conditions) was tested by adding culture medium to the desiccated strains and maintaining them at room temperature (22–24 °C) for five months. Survival was measured as retention, partial or total loss of pigmentation as previously described [22,35,52] and microscopic observation of the cultures.

## 3. Results

### 3.1. Physicochemical Characteristics of Soils

Physicochemical soil features are depicted in Table 1. According to the Baize standards for the limestone content [53], the two soils were considered moderately calcareous. Both soils showed relatively high gypsum content. According to the Barzanji scale [54], the Taleb Larbi soil belongs to the slightly gypsiferous class, and the Chott Kralla soil belongs to the medium gypsiferous class. The organic matter content of the two sites was very low; likewise, low carbon and nitrogen contents were also recorded. Both soils showed high electrical conductivity and high salinity but with differences between them; according to the salinity scale of Aubert [55], these values lead us to classify the Taleb Larbi soil as slightly saline and the Chott Kralla soil as very saline. Therefore, the samples from Chott Kralla were named saline biocrusts (SBC) and those from Taleb Larbi, less saline biocrusts (LSB).

### 3.2. Biocrust Characteristics

**Figure 2:**
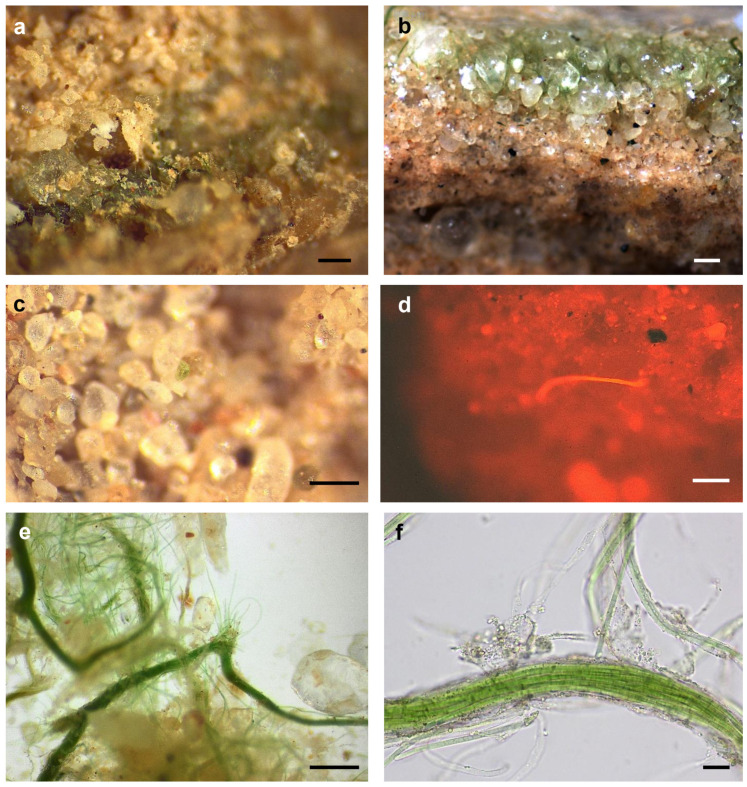
Microphotographs of the biocrusts samples. (**a**) Dry and (**b**) wet biocrust from Taleb Larbi (LSB, Less saline biocrusts)), (**c**,**d**) Dissecting microscopy of biocrusts from Chott Kralla (SBC, Saline biocrusts), (**c**) bright field microscopy, (**d**) autofluorescence microscopy, (**e**,**f**) Bundels in biocrusts from Taleb Larbi (LSB). Scale bar 200 μm, except in (**f**) 20 μm.

Macroscopic inspection of the LSBs from Taleb Larbi showed a continuous green layer several millimetres below the soil surface with bundles or filaments disposed perpendicular to the surface (Figure 2a), which migrated to the surface when the soil was wetted (Figure 2b) in relation to the movements shown for some cyanobacteria in response to wetting events in biological soil crusts [56]. In contrast to the high amount of biomass perceptible to the eye in the LSB samples, in the Chott Kralla SBC samples, it was very difficult to find cyanobacterial biomass, although several green areas were detected under stereomicroscopy (Figure 2c), and some filaments or bundles were also observed due to phycocyanin autofluorescence (Figure 2d). When LSB samples were inspected under the microscope, a high number of bundles was observed, corresponding to *Microcoleus* spp., together with motile filaments migrating from the bundles (Figure 2e,f).

### 3.3. Morphological and Molecular (16S rRNA Gene) Analysis of Cultures: Polyphasic Identification of Isolated Strains

**Figure 3:**
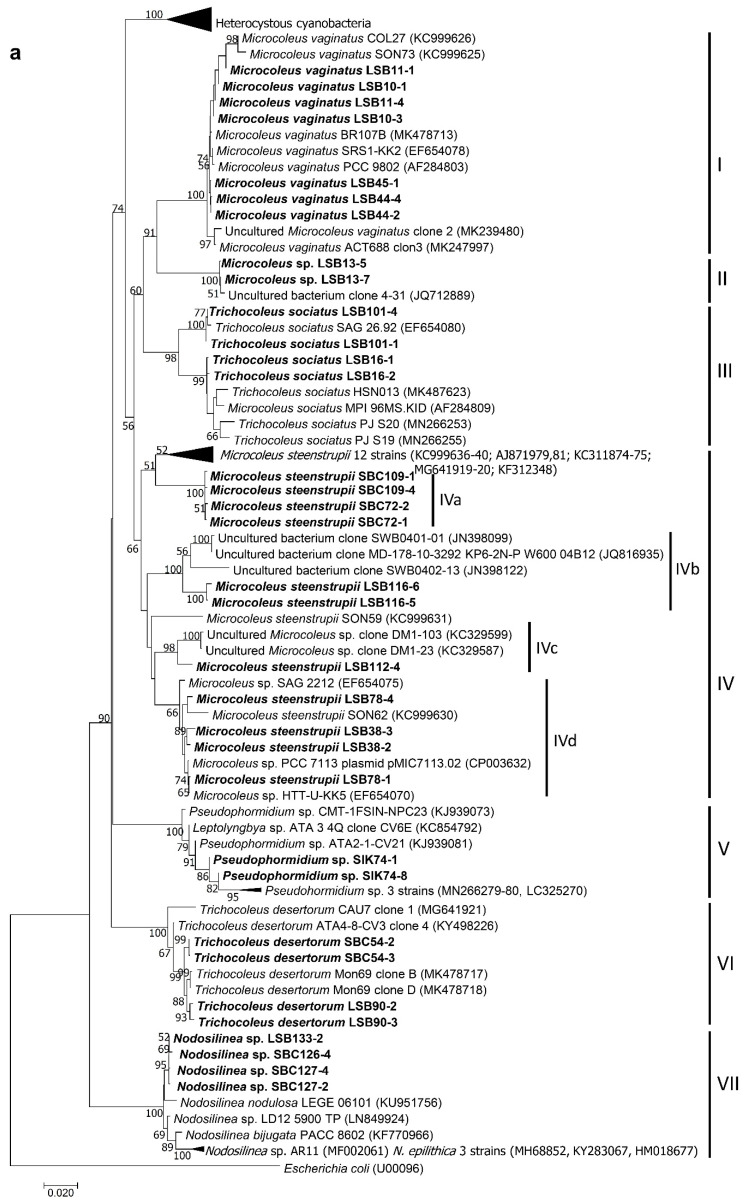

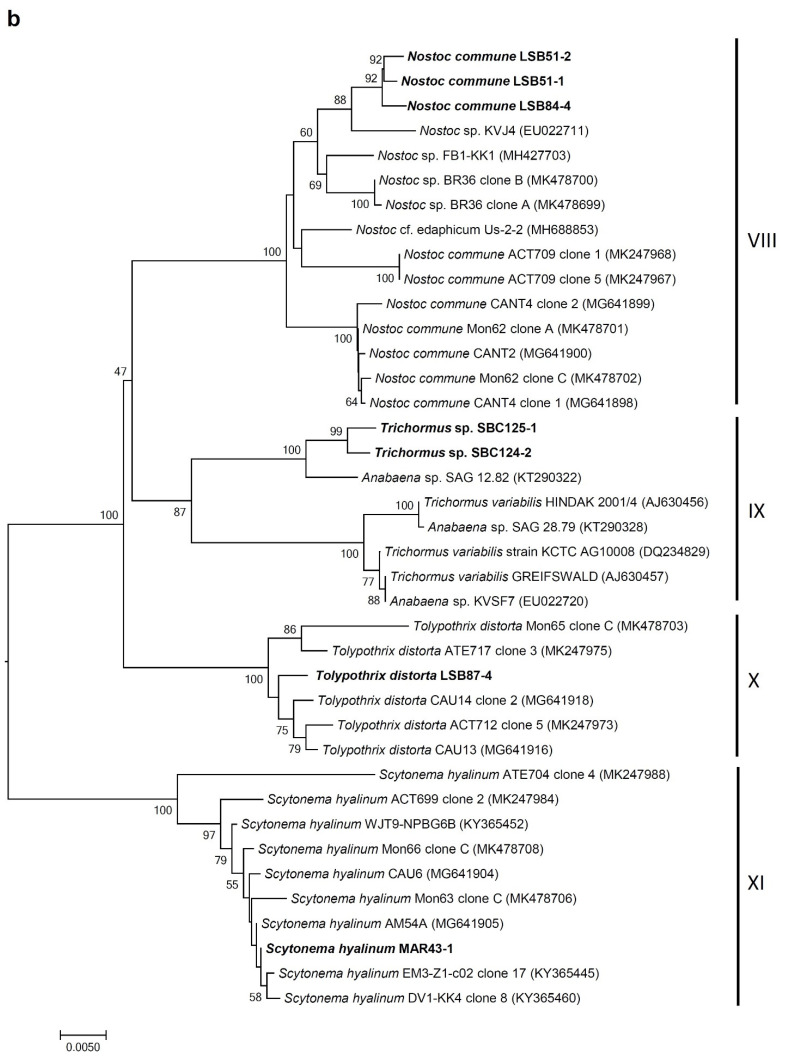
(**a**) Phylogenetic tree obtained by the neighbor-joining method based on the analysis of the 16S rRNA gene, showing the position of the sequences of the isolated strains obtained in the present study (in bold). (**b**) heterocystous cyanobacteria expanded. Numbers near node indicate bootstrap values greater than or equal to 50.

**Figure 4:**
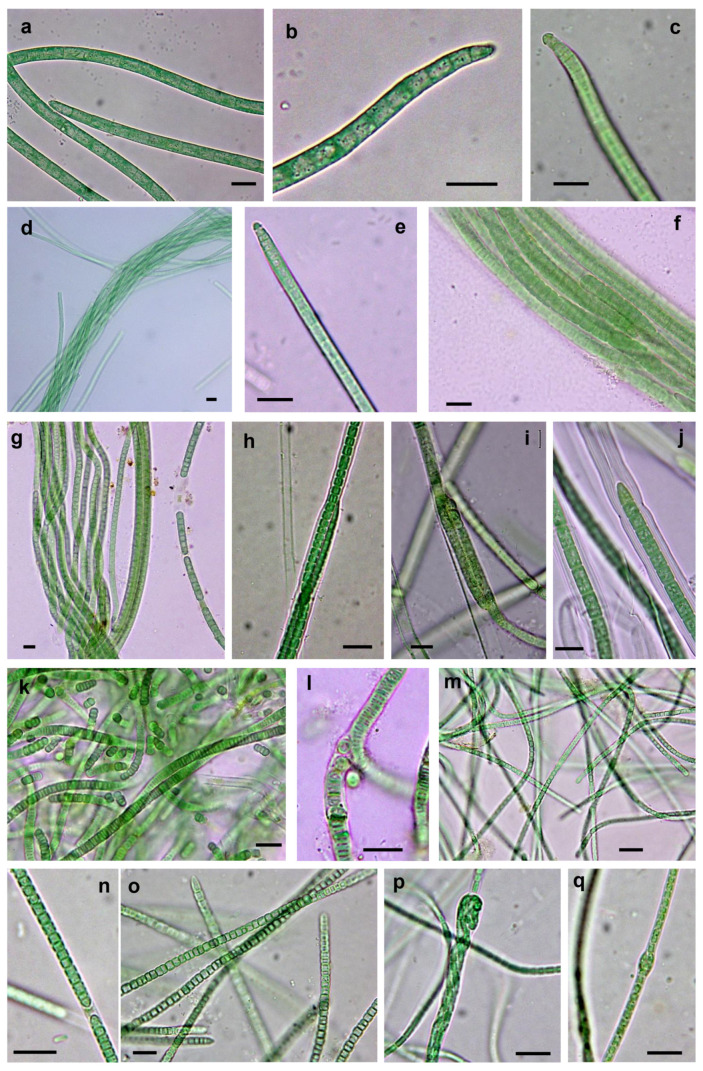
Microphotographs of non-heterocystous cyanobacterial strains. (**a**,**b**) *Microcoleus vaginatus* LSB45, (**c**) *M. vaginatus* LSB11, (**d**,**e**) *Microcoleus* sp. LSB13, (**f**,**g**) *Trichocoleus sociatus* LSB101, (**h**) *Microcoleus steenstrupii* SBC72, (**i**) *M. steenstrupii* LSB116, (**j**) *M. steenstrupii* LSB112, (**k**) and (l) *Pseudophormidium* sp. SIK74 (**m**,**n**) *Trichocoleus desertorum* SBC54, (**o**) *T. desertorum* LSB90, (**p**) *Nodosilinea* sp. SBC126, (**q**) *Nodosilinea* sp. LSB127. Scale bar 10 µm.

**Figure 5:**
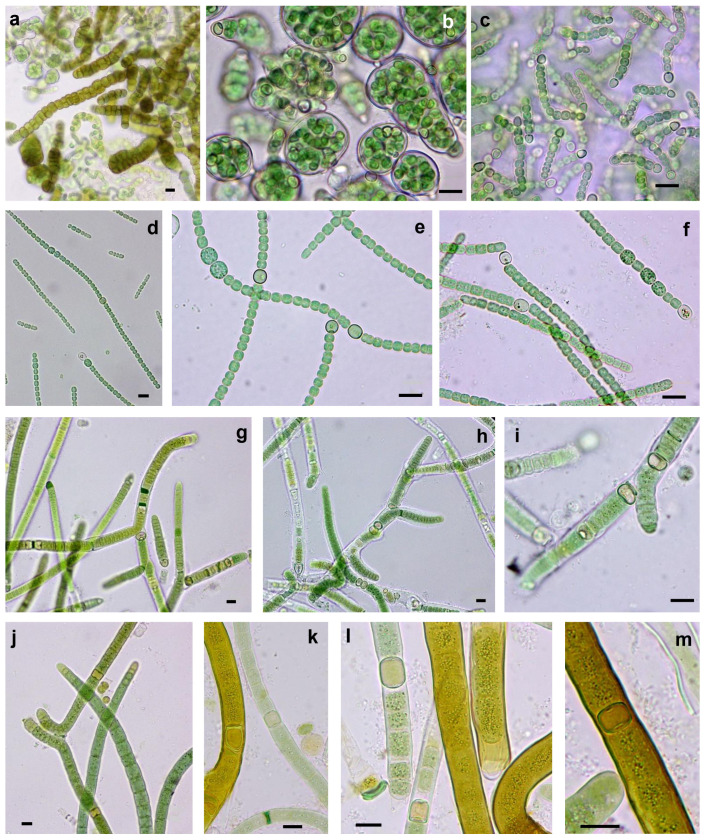
Microphotographs of heterocystous cyanobacterial strains. (**a**,**b**) *Nostoc commune* LSB51, (**c**) *N. commune* LSB84, (**d**,**e**) *Trichormus* sp. SBC124, (**f**) *Trichormus* sp. SBC125, (**g**–**i**) *Tolypothrix distorta* LSB87, (**j**–**m**) *Scytonema hyalinum* MAR43. Scale bar 10 µm.

After the isolation, culture and characterization processes, we obtained 23 strains, including 16 from the Taleb Larbi site (LSB strains) and seven from the Chott Kralla site (SBC strains) (Figure 3, Figure 4 and Figure 5, Table 2). Five strains were heterocystous cyanobacteria, and the remaining 18 strains were filamentous non-heterocystous cyanobacteria. Most of the non-heterocystous strains were bundle-forming cyanobacteria (*Microcoleus* and *Trichocoleus* species), and three strains were assigned to the *Nodosilinea* genus. In addition, two strains (*Scytonema hyalinum* and *Pseudophormidium* sp.) previously isolated from the Algerian Sahara Desert, not far from Chott Kralla, but isolated from different microhabitat (endolithic and hypolythic, respectively), were also analysed, since both were important biocrust components from this location (see the cyanobacterial community composition section below). Figure 3 represents the phylogenetic position of the 16S rRNA sequences of the isolated strains, while Figure 4 and Figure 5, and Table 2 show the morphological features of these strains, the assigned culture collection number and the isolation source.

#### 3.3.1. Non-Heterocystous Strains

The 16S rRNA gene sequences of four strains (LSB10, LSB11, LSB 44 and LSB 45) identified as *Microcoleus vaginatus* and isolated directly by bundle micromanipulation from Taleb Larbi grouped in the phylogenetic tree in cluster I with known representatives of *M. vaginatus* from soils (Figure 3 and Figure 4a–c, Table 2). The similarity within this group ranged from 98.9 to 100%. The morphological characteristics were very similar, with filaments not forming bundles in culture, with ends with sometimes forming calyptra (Figure 4c) and sizes compatible with *M. vaginatus* (Table 2). The LSB13 strain was also directly isolated from Taleb Larbi bundles and fell on the phylogenetic tree in cluster II with uncultured cyanobacteria (Figure 3a). This cluster, although sister to the *M. vaginatus* cluster and with a very good bootstrap, had a similarity of only 91.2–93.2%. The morphological characteristics of this strain were also similar to those of *M. vaginatus* (Figure 4d,e), but with slightly smaller cellular sizes (Table 2) and the peculiarity of forming bundles in culture (Figure 4d), so it was identified as *Microcoleus* sp.

The strains LSB16 and LSB101, isolated from Taleb Larbi, were mapped in cluster III, corresponding to *Trichocoleus sociatus* (Figure 3a). They presented solitary, unbranched filaments, colourless sheaths open at the ends, sometimes containing 8–13 or more trichomes, spirally coiled or entangled; the cells were nearly square (Table 2), slightly constricted and rounded at the apex (Figure 4f,g).

Six strains with morphological characteristics compatible with *Microcoleus steenstrupii* were grouped in cluster IV, which included *Microcoleus* sp. and *M. steenstrupii* sequences. As previously described [35], this group is morphologically and phylogenetically heterogeneous (Figure 3 and Figure 4h–j, Table 2), with sequence similarities ranging from 90% to 100%, and needs taxonomic revision, so it can be divided into several clear subclusters (Figure 3a).

The *Pseudophormidium* sp. isolate fell in cluster V, together with other representatives of this genus, with typical phenotypic characteristics, such as filaments showing false branches and trichomes often disintegrated into short numerous segments. Sheaths were thin and colourless, with trichomes constricted at cross walls, and the cells were distinctly shorter than they were wide (Figure 3 and Figure 4k,l).

Two strains, one isolated from Chott Kralla (SBC54) and the other from Taleb Larbi (LSB90), were identified as *Trichocoleus desertorum*, as they were placed in cluster VI in the phylogenetic tree with other *T. desertorum* strains (Figure 3a) and showed typical morphological traits from this taxon. The cells were typically wider than they were long (Table 2) and formed thin trichomes alone or in bundles surrounded by a colourless sheath. Terminal cells were rounded or conical without calyptra, and the cells were slightly constricted, with some harbouring inclusions (Figure 4m–o). The strain isolated from the saline biocrust Chott Kralla (SBC54) was slightly thinner than that isolated from Taleb Larbi (LSB90) (Table 2 and Figure 4m–o).

The rest of the filamentous non-heterocystous cyanobacterial strains did not form bundles in nature. They belong to the *Nodosilinea* genus. Two strains were isolated from Chott Kralla (SBC126 and SBC127), and one was isolated from Taleb Larbi (LSB133). They presented thin unbranched, or rarely pseudobranched, filaments, that sometimes formed nodes enclosed in thin transparent sheaths and with cells normally longer than they were wide (Table 2, Figure 4p,q). The 16S rDNA sequences were placed in the phylogenetic tree in cluster VII with other *Nodosilinea* spp. from the NCBI database (Figure 3a).

#### 3.3.2. Heterocystous Strains

Two *Nostoc commune* strains were isolated from the Taleb Larbi site (LSB51 and LSB84) with morphological characteristics and sizes typical of *N. commune*: almost spherical cells, entangled trichomes that were observed either individually or together and surrounded by a clearly visible sheath with the heterocyst outside the sheath (Table 2, Figure 5a–c). The 16S rDNA sequences were very similar and mapped in the phylogenetic tree in cluster VIII with other sequences of *N. commune* isolated from soils (Figure 3b).

Two strains isolated from Chott Kralla, SBC124 and SBC125, mapped in cluster IX, together with *Trichormus* and *Anabaena* sequences downloaded from the NCBI database (Figure 3b). Species of the genera *Trichormus* and *Anabaena* are morphologically similar, but the strategy of akinete formation is completely different [37]; in *Anabaena*, akinetes develop next to heterocysts or slightly distant from them, while in *Trichormus*, akinetes start to develop more or less in the middle between two distant heterocysts, as found in our cultures (Figure 5d–f). Therefore, these strains were identified as *Trichormus* sp. They had filaments without sheaths or gelatinous envelopes and trichomes that were cylindrical, irregularly coiled, constricted at the cross walls, and not attenuated or slightly narrowed at the ends. The cells were barrel-shaped, and the terminal cells were rounded or conical-rounded. Heterocysts were intercalary, solitary, nearly spherical or slightly elongated. Akinetes were oval, longer than they were wide, with a smooth, colourless to yellow-brown cell wall. *Trichormus* sp. reproduce by hormogonia and by the germination of akinetes.

A *Tolypothrix distorta* strain (LSB87) was isolated from Taleb Larbi, with typical false branched filaments, usually with a heterocyst in the base of the branch (Figure 5g–i), which fell in the well-supported cluster X with other *T. distorta* strains (Figure 3b).

The strain of *S. hyalinum* also showed typical characteristics of the genus (Figure 5j–m), and its sequence was placed in a cluster (cluster XI) of known representatives of *S. hyalinum* from biocrusts (Figure 3b).

### 3.4. Molecular Analysis of Cyanobacterial Community Composition

Good’s coverage estimates reached 99.99% in both samples, indicating that the majority of the cyanobacterial diversity was captured. The rarefaction curve also indicated that the number of observed OTUs for LSB samples was higher than for SBC samples (Figure S1).

**Figure 6:**
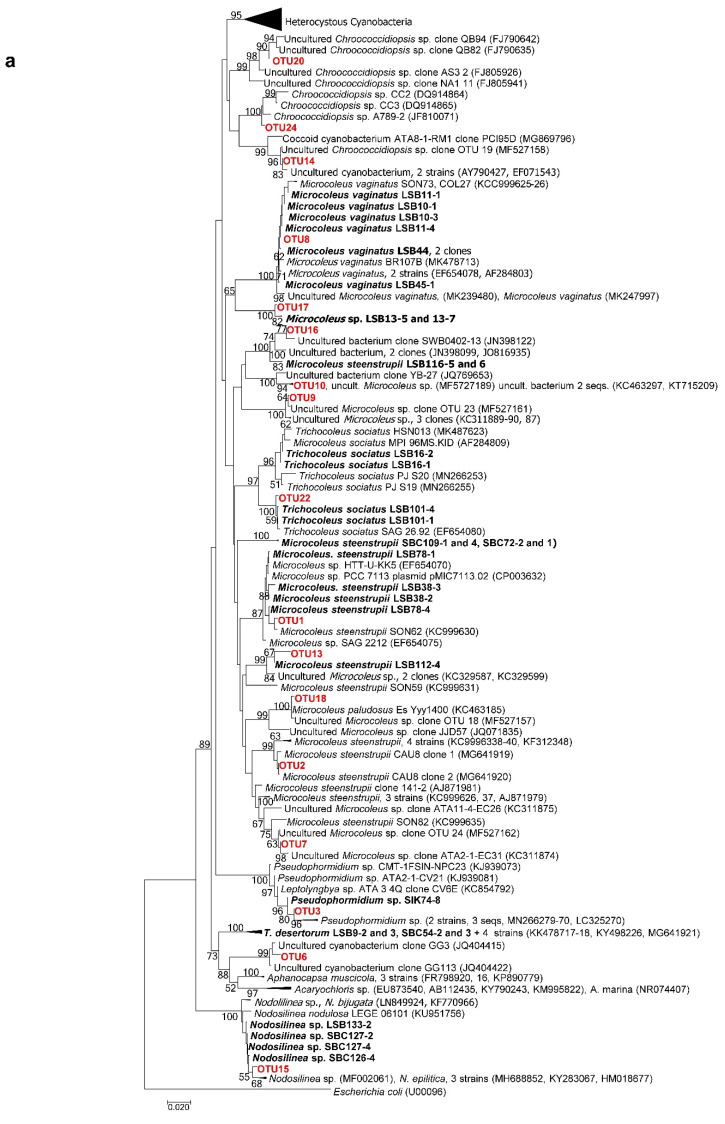

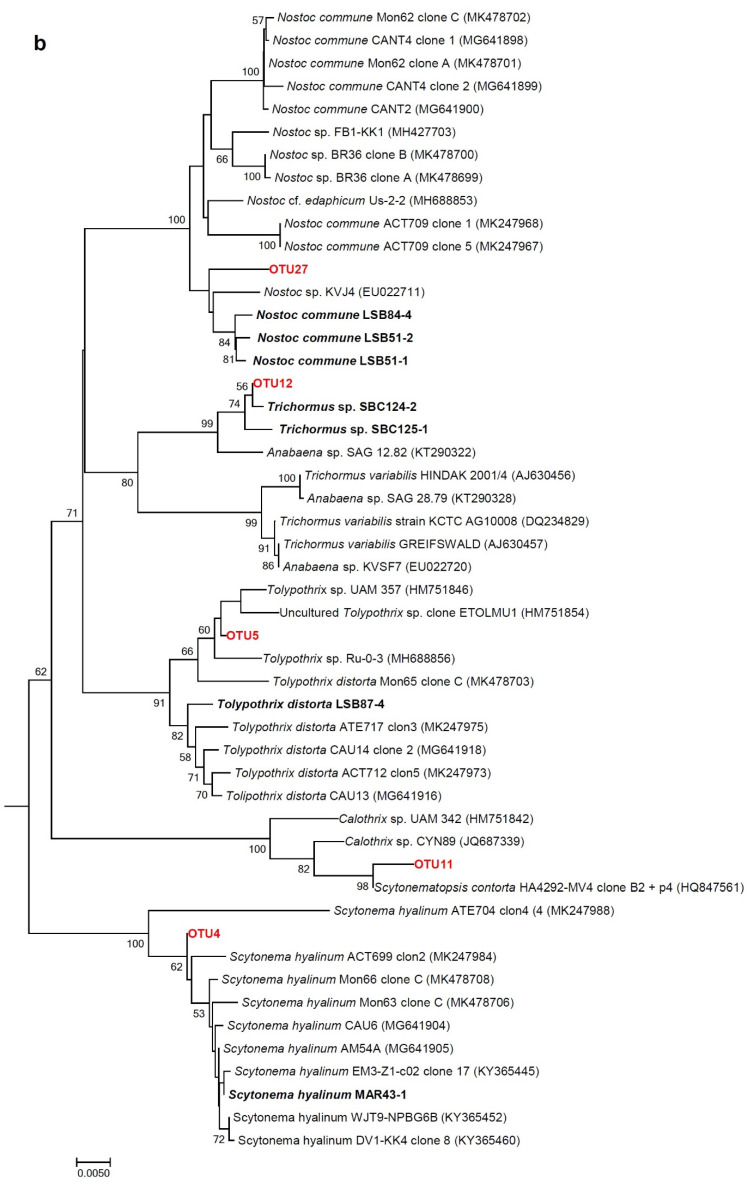
(**a**) Phylogenetic tree obtained by the neighbor-joining method based on the analysis of 16S rRNA gene, showing the position of the sequences of the isolated strains obtained in the present study (in bold) and the Operational Taxonomic Units (OTUs) determined by amplicon metagenomics (in red). (**b**) heterocystous cyanobacteria expanded. Numbers near node indicate bootstrap values greater than or equal to 50.

**Figure 7:**
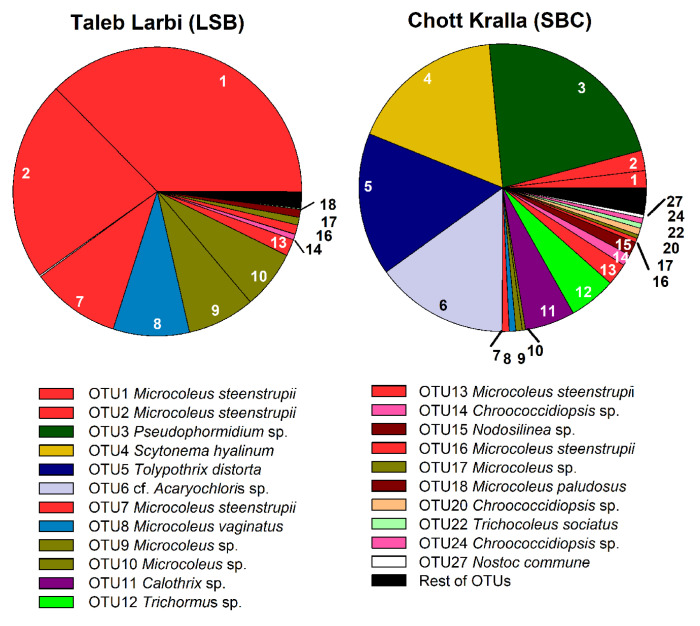
Cyanobacterial community composition from the studied biocrusts. Each taxon is represented by a different color, and each OTU by a number (see Figure 6 for OTU numbers and taxonomic assignments). SBC, Saline biocrusts; LSB, Less saline biocrusts.

Figure 6 shows the phylogenetic tree in which the obtained OTUs, together with the isolated strain sequences and similar sequences downloaded from the database, were included, which allowed the taxonomic assignments displayed in Figure 7, along with the relative abundance of the OTUs in each site. Of the 22 main OTUs, 13 corresponded to sequences of isolated cultures, which, in general, were the most abundant cyanobacteria in the locations, such as *M. steenstrupii*, *M. vaginatus*, *Microcoleus* spp., *Pseudophormidium*, *Scytonema hyalinum* and *Tolypothrix distorta* (Figure 6 and Figure 7)

In Taleb Larbi, the less saline biocrust, all the analysed OTUs were filamentous non-heterocystous cyanobacteria, except OTU14, assigned to unicellular *Chroococcidiopsis* sp., which was found in low abundance (1%). At this site, the three most abundant OTUs, accounting for 69.8%, corresponded to *M. steenstrupii* (Figure 7). *M. vaginatus* (OTU8) and two OTUs of *Microcoleus* sp. (OTUs 9 and 10) were also present in similar proportions (6–8% each). Two other *M. steenstrupii* OTUs, OTU13 (2%) and 16 (1%), were present in lower proportions, and the other OTUs with relative abundances of approximately 1% corresponded to *Microcoleus* sp. (OTU17) and *M. paludosus* (OTU18) (Figure 7).

The cyanobacterial community composition in Chott Kralla, the saline biocrust, was more diverse and had a high abundance of heterocystous cyanobacteria (Figure 7). The most abundant OTUs corresponded to the filamentous non-heterocystous *Pseudophormidium* (OTU3), with a relative abundance at this site of 22%. It was followed by the heterocystous cyanobacteria *S. hyalinum* (OTU4) and *T. distorta* (OTU5) and the unicellular OTU6, identified as cf. *Acaryochloris*, with relative abundances of approximately 15% each. Two other heterocystous cyanobacteria, *Calothrix* (OTU11) and *Trichormus* (OTU12), were present, with relative abundances of approximately 5% each, while three OTUs assigned as *M. steenstrupii* (OTUs 1, 2 and 13) showed relative abundances between 2 and 2.5%. Other OTUs with relative abundances between 0.5 and 1.5% included *Microcoleus* spp. (OTUs 9, 10, and 17), *M. steenstrupii* (OTUs 7 and 16), *M. vaginatus* (OTU8) *Chroococcidiopsis* spp. (OTUs 14, 20, and 24), *Nodosilinea* sp. (OTU15), *T. sociatus* (OTU22) and *N. commune* (OTU27).

### 3.5. Resistance of Strains to Extreme Heat and Desiccation

**Figure 8:**
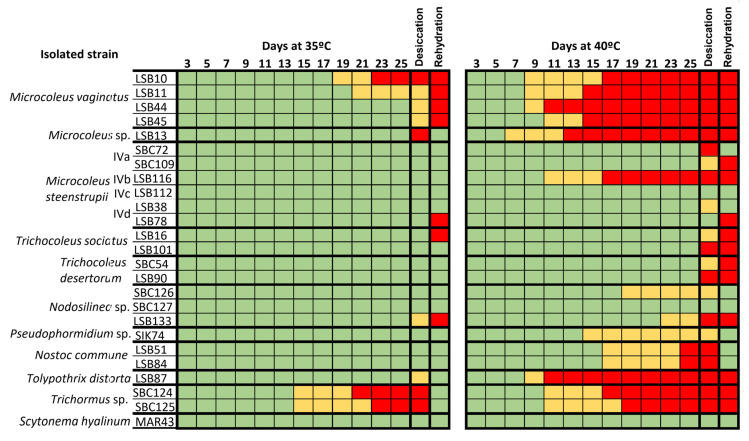
Survival of cyanobacterial cultures over 25 days at 35 and 40 °C, subsequent response of cultures to two months-long desiccation treatment at 35 and 40 °C, and later survival following the addition of culture medium to the desiccated cells and maintained at room temperature. Green-coloured rectangles indicate survival, orange-coloured rectangles indicate some deterioration with initial loss of pigmentation, and red-coloured rectangles indicate death.

The ability of the isolated strains to survive extreme temperatures (35 and 40 °C) and desiccation, was tested two ways: the resistance (the ability of the cyanobacteria to withstand the extreme conditions), and the resilience (the ability of the cyanobacteria to recover following the extreme conditions, after rehydration with culture medium at room temperature) (Figure 8). Differences were found regarding the responses at 35 and 40 °C. The majority of the strains resist 35 °C except the two *Trichormus* sp. strains, although they were resilient as they recovered well after rehydration. However, *M. vaginatus* strains did not resist desiccation at 35 °C and did not survive after rehydration. In contrast, at 40 °C, the majority of strains neither survived nor resisted desiccation, but some species, such as *S. hyalinum*, *M. steenstrupii*, *Pseudophormidium* sp., *N. commune* or *Nodosilinea*, were resilient and recovered after rehydration (Figure 8).

## 4. Discussion

Biocrusts of the topsoil of the Sahara Desert are exposed to extreme conditions, such as high ultraviolet light irradiation, large changes in temperatures and low moisture availability, including long periods of desiccation. In addition to these hyperarid conditions, some locations in the desert, such as the studied locations, are saline or even hypersaline, such as the dry salt lake Chott Kralla, making this zone of the Sahara Desert an exceptional polyextreme environment. These polyextreme environmental conditions influence the structure of existing cyanobacterial communities in biocrusts, since only those able to withstand these hostile conditions will live under such stress; therefore, cyanobacteria from these biocrusts and experiencing these conditions must be polyextremophiles or polyextremotolerants.

Our results show a clear dominance of the bundle-forming *Microcoleus* spp. (96.4%) in the less saline soils of the Taleb Larbi location, with a high abundance of 72.8% *M. steenstrupii*, as opposed to the low amount of *M. vaginatus* (8.6%) and other *Microcoleus* spp. found at 15% abundance. However, in the biocrust of the dry salt lake Chott Kralla, heterocystous cyanobacteria dominated, with an abundance of 44.4%, although the most abundant at this site was the non-bundle cyanobacteria *Pseudophormidium* sp. (22%), and the unicellular cf. *Acaryochloris* was also found in high amounts (15%).

Differences in the cyanobacterial community composition of biocrusts have been found depending on the geographically studied region. *Leptolyngbya* and *Porphyrosiphon* were dominant in subtropical biocrusts [57], and *Leptolyngbya* and *Trichocoleus* were dominant in crusts from degraded soils [24]. *Microcoleus* spp. dominance in the less saline biocrusts was similar to observations in North American and Mediterranean semiarid crusts [23,35], where *M. steenstrupii* was more abundant in hot environments, as was found in this study. However, the high abundance of heterocystous cyanobacteria found in the dry salt lake was unexpected but similar to that found in a semidesert of Central Mexico, with high temperatures and high insolation all year round [22].

Cyanobacteria have been considered ubiquitous for a long time since they can be found in almost every ecosystem, from polar regions to thermal habitats [58]. However, as previously stated, not all cyanobacterial taxa are ubiquitous, and specific ecophysiological traits allow them to occupy distinct ecological niches, which explains their differential geographical distribution [38,59]. In turn, changes in cyanobacterial dominance depending on the microenvironmental conditions, even within the same area, have been described [22,60,61], as in this study.

Temperature has been recently found to be the principal driver in the biogeographical distribution of cyanobacterial communities in biocrusts [22,23,35,57], whereby some species, such as *M. steenstrupii* and *S. hyalinum*, were dominant in hot locations, in agreement with our results. Previous results on the effect of different temperatures on cyanobacterial strains isolated from biocrusts from different deserts showed the optimal growth rates ranging from 18 to 30 °C, although some cyanobacteria also grew or survive at higher temperatures, showing tolerance to these temperatures. Then, these cyanobacteria can be considered thermotolerant rather than thermophilic [23,35]. However, as previously stated, the studied locations not only experience high temperatures but also a combination of different stressors. The polyextreme environmental conditions found in the studied sites exert selective stresses on cyanobacteria, and consequently, several adaptive mechanisms and/or protection strategies have evolved to also face high solar radiation, repeated cycles of desiccation and rewetting and/or prolonged desiccation, in addition to salt stress.

Cyanobacteria are among the most successful organisms in saline environments, and the existence of a wide range of halophilic and halotolerant species has long been recognized [62]. The production of extracellular polymeric substances (EPS) has been implicated in a salt-buffering effect, increasing tolerance in salt-stressed cells [63,64]. It has been reported that under salt stress, the amount of EPS increased up to 65% of dry weight in a terrestrial *Nostoc* sp. [65]. The accumulation of organic osmolytes, such as sucrose and trehalose, plays another important role in salinity tolerance [62,63]. The results from this study show clear differences in the communities depending on the salinity of the studied soils in that a dominance of heterocystous cyanobacteria was found in the more saline soils, in contrast to non-heterocystous cyanobacteria dominance in the less saline soils, in agreement with previous studies that showed a high tolerance of Nostocales to different ranges of salinity in soils [66,67,68]. However, halophilic cyanobacteria generally belong to Oscillatoriales and unicellular cyanobacteria, although, notably, the majority of studies were carried out in water-related environments such as hypersaline lakes, lagoons, springs or seawater habitats [62]. In addition, the distinction between halophilic and halotolerant cyanobacteria should be noted, in which halophilic cyanobacteria are distinguished by their requirement of high salt concentrations for growth, while halotolerants are those that can grow in the presence and absence of high concentrations of salt [69]. Therefore, the presence of the cyanobacteria found in our study area, which were also widely distributed in other non-saline geographic locations around the world [70], indicates that they are halotolerant rather than truly halophilic.

Desiccation effects are closely related to salinity, in that both stressors reflect two different forms of water deprivation, and water availability is strongly influenced by salinity. The combination of both stressors leads to strong cellular dehydration. A number of cyanobacteria are known to have the ability to survive under severe drought conditions, and different mechanisms to protect cells against possible resulting damage are involved ([71], and references therein). Long-standing studies on the survival of *Nostoc* spp., after decades of desiccation, have shown that cells can resume physiological activities within minutes of wetting [72,73], being able to develop protective mechanisms to avoid damage after prolonged cell desiccation [74]. Natural populations of *M. vaginatus* in biocrusts rapidly recover photosynthetic activity following hydration after desiccation, and a rapid induction of genes related to the response to desiccation stress, such as genes for oxidative stress and genes for exopolysaccharide synthesis, has also been found [3]. Recent studies on *Leptolynbya ohadii*, as a model organism of filamentous cyanobacteria inhabiting desert biocrusts, showed quick resumption of photosynthesis upon rewetting in contrast to a slow change in the transcript profile, suggesting that, during dehydration, in addition to preparing for dehydration, the cells also prepare for a forthcoming rewetting [75].

Dehydration severely damages membrane structures, proteins, and nucleic acids, primarily because of oxidative stress resulting from the release of reactive oxygen species (ROS) during desiccation and is lethal to most organisms [76]. Therefore, cyanobacteria able to survive desiccation have developed different strategies, as explained above, but a general strategy seems to be the ability to survive inactively during desiccation and recover rapidly after desiccation. The deceleration of vital activity or near complete inhibition of metabolic activity, known as *anhydrobiosis* [77], allows cells to undergo nearly absolute dehydration during air-drying without being killed; therefore, the cells enter a dormancy state at desiccation onset and resume metabolic activities when water becomes available ([78], and references therein). In turn, the capacity for desiccation tolerance is linked to the presence of a sheath and mucilage, which can help protect cells against physical desiccation. A large number of functions have been ascribed to EPS in relation to protection against desiccation; these include the retention of large amounts of water and the formation of a gel that stabilizes the macromolecular components, large amounts of secreted acidic water stress protein A and highly stable and active superoxide dismutase, elimination of release of ROS for protection from DNA breakage and lipid peroxidation, and inhibition of fusion of membrane vesicles [79,80]. In relation to this protective mechanism, it should be noted that the species recorded in our study showed thick layers of extracellular mucilage due to the excretion of EPS, which allows them to overcome long periods of drought.

Regarding the high levels of ultraviolet radiation (UVR), *Microcoleus* spp. have the ability to undergo vertical migrations from the surface to an underground shelter, avoiding increased light intensities [81]. However, sessile heterocystous genera, such as those found in this study, *Scytonema*, *Tolypothrix*, *Calothrix* and *Nostoc*, which live on the topsoil, resort to the production of large quantities of sunscreen pigments, such as the lipid soluble yellow-brown pigment scytonemin, immobilized on EPS, and other water-soluble UV radiation-absorbing pigments, protecting the cytosolic components from photodegradation [81,82]. Microbial communities that inhabit halite substrates in the hyperarid Atacama Desert show a resilient evolutionary and adaptive strategy, increasing scytonemin biosynthesis in response to increases in UV-A irradiations [83,84]. Scytonemin is possibly even more important in UVR protection, when cyanobacteria are exposed to desiccation, since alternative UVR coping mechanisms are inactive, and damage could increase [85]. Nevertheless, scytonemin accumulation significantly decreases soil albedo, increasing surface temperature, which, in turn, induces the replacement of thermosensitive species with thermotolerant forms [86]. Our results show a high abundance of *Pseudophormidium* sp. in this type of dark-pigmented heterocystous crust in relation to its tolerance to high temperatures and dehydration in thermodesiccation bioassays. This result is also in agreement with previous records of *Pseudophormidium* in soils from extreme environments, such as the hyperarid Atacama Desert [87,88,89], and as a water deficiency-resistant cyanobacterium found in steppes of the South Ural region [90].

In addition to *Pseudophormidium* sp., experimental results from this study confirm that some other species, such as *S. hyalinum*, *N. commune*, *M. steenstrupii* and *Nodosilinea* sp. are resistant to extreme heat and desiccation in relation to their high abundance and/or dominance in the hyperarid studied sites. Coccoid cyanobacteria, such as *Acaryochloris* and *Chroococcidiopsis*, have also been found in extreme environmental conditions worldwide [91,92,93,94,95], demonstrating a multiple tolerance [96]. However, it should be noted that *Acaryochloris* spp., from hot or cold arid deserts, have been reported as endolithic species that live inside rocks as refuges [97,98], and *Chroococcidiopsis* spp. are typically considered hypolythic and endolithic organisms [96], although they have also been reported in biocrusts [22,23]. Thus, their occurrence in the Sahara desert topsoil biocrusts may further support their extremotolerance. However, what is striking is the high abundance of *T. distorta* found in the dry salt lake Chott Kralla, since previous results as well as experimental results from this study showed no thermotolerance for this species [22,35,99]. A possible explanation for this result could be related to the development of cell survival stages, akinetes, typically found, for instance, in *Nostoc* spp., but not well documented for *Tolypothrix* species, although they have been reported for *T. distorta* [100] and other species of this genus [36,101]. The ability to develop akinete-like cells could allow dormancy during extreme heat and desiccation, germination and development of new filaments when conditions become suitable, and, therefore, resilience and recovery from polyextreme environmental conditions when periodic environmental changes allow growth. A recent study found *T. distorta* from arid biocrusts resilient to extreme conditions of degraded soils [24]. Therefore, some cyanobacteria living in these polyextreme conditions can resist extreme conditions, while others are resilient and can be dormant and recover following these extreme conditions. Our findings contribute to our understanding of the effect of the variation in microenvironmental conditions on the cyanobacterial community composition in biocrusts from hyperarid deserts, which provides valuable information for assessing ecosystem functioning and development in these polyextreme environments.

## Figures and Tables

**Table 1:** Physicochemical soil properties. (SBC, Saline biocrusts; LSB, Less saline biocrusts).

Soil Characteristic	Site	
	Taleb Larbi (LSB)	Chott Kralla (SBC)
pH	7.44	7.51
EC _1:5_ (dS·m^−1^)	1.76	3.28
Salinity (%)	1.99	3.71
Total limestone (%)	9.81	11.16
Gypsum (%)	7.82	13.76
Organic matter (%)	0.58	0.45
Organic C (%)	0.34	0.26
Total N(%)	0.042	0.019

**Table 2:** Morphological characteristics of cyanobacterial cells. Measurements are given as mean ± standar deviation/range, *n* = 100. * Isolated from bundles. (SBC, Saline biocrusts; LSB, Less saline biocrusts).

Taxon		Strain	Culture Collection N°	Sampling Site	Type of Cell	Breath (µm)	Length (µm)
*Microcoleus vaginatus*		LSB45 *	UAM 886	Taleb Larbi	Vegetative cells	3.6 ± 0.3/2.9–4.1	4.4 ± 0.9/2.6–6.9
		LSB44 *	UAM 887	Taleb Larbi	Vegetative cells	3.8 ± 0.3/3.2–4.5	3.9 ± 0.7/2.8–5.6
		LSB10 *	UAM 888	Taleb Larbi	Vegetative cells	5.1 ± 0.4/4.3–5.8	3.9 ± 0.5/2.6–5.9
		LSB11 *	UAM 889	Taleb Larbi	Vegetative cells	5.5 ± 0.5/4.7–6.8	4.4 ± 0.6/3.2–5.9
*Microcoleus* sp.		LSB13 *	UAM 885	Taleb Larbi	Vegetative cells	4.8 ± 0.6/3.5–5.9	3 ± 0.6/2.1–4.6
*Microcoleus steenstrpii*	Cluster IVa	SBC109	UAM 872	Chott Kralla	Vegetative cells	2.7 ± 0.2/2–3.7	3.9 ± 0.6/2.7–5.9
		SBC72	UAM 873	Chott Kralla	Vegetative cells	3.6 ± 0.4/2.8–4.4	3.6 ± 0.4/2.7–4.6
	Cluster IVb	LSB112 *	UAM 874	Taleb Larbi	Vegetative cells	5.3 ± 0.4/4.3–6.4	8 ± 1.3/5.1–11.2
	Claster IVc	LSB116 *	UAM 877	Taleb Larbi	Vegetative cells	6.1 ± 1.1/4.2–8.6	5.6 ± 0.9/3.5–8.1
	Cluster IVd	LSB78 *	UAM 875	Taleb Larbi	Vegetative cells	6.8 ± 0.5/5.3–7.7	4.6 ± 0.6/3.4–6.7
		LSB38 *	UAM 876	Taleb Larbi	Vegetative cells	6 ± 0.9/4.4–8.4	6.3 ± 1.2/4–9.4
*Trichocoleus sociatus*		LSB16	UAM 883	Taleb Larbi	Vegetative cells	5.2 ± 0.2/4.6–5.8	3.3 ± 0.4/2.6–4.3
		LSB101	UAM 884	Taleb Larbi	Vegetative cells	4.5 ± 0.8/3.2–7.1	4.2 ± 0.7/3.2–6.1
*Trichocoleus desertorum*		SBC54	UAM 890	Chott Kralla	Vegetative cells	2.8 ± 0.2/2.4–3.3	2.8 ± 0.4/2–3.6
		LSB90	UAM 891	Taleb Larbi	Vegetative cells	3.9 ± 0.3/3.2–4.5	3 ± 0.5/2.2–4.5
*Nodosilinea* sp.		SBC127	UAM 892	Chott Kralla	Vegetative cells	2 ± 0.2/1.6–2.6	2.5 ± 0.3/1.7–3.3
		SBC126	UAM 893	Chott Kralla	Vegetative cells	2.1 ± 0.3/1.4–2.8	3.6 ± 0.5/2.4–5
		LSB133	UAM 894	Taleb Larbi	Vegetative cells	2 ± 0.3/1.4–2.9	3.1 ± 0.6/2–4.3
*Nostoc commune*		LSB51	UAM 881	Taleb Larbi	Vegetative cells	4.4 ± 0.8/3.2–6.2	4.3 ± 0.9/2.9–6.8
					Heterocysts	3.9 ± 0.4/3.1–5.1	4.5 ± 0.8/3.2–6.6
		LSB84	UAM 882	Taleb Larbi	Vegetative cells	3.7 ± 0.3/3–4.5	3.4 ± 0.5/2.3–5.1
					Heterocysts	3.8 ± 0.3/3–4.7	4.5 ± 0.5/3–5.8
*Tolypothrix distorta*		LSB87	UAM 878	Taleb Larbi	Vegetative cells	9.7 ± 1/7.4–12.3	4.3 ± 0.7/2.6–6.2
					Intercalary Heterocysts	9.7 ± 0.7/8.3–11.6	9.9 ± 1.3/7–13.5
					Terminal Heterocysts	9.5 ± 1.1/7.6–12	9.7 ± 0.7/8.5–10.8
*Trichormus* sp.		SBC124	UAM 879	Chott Kralla	Vegetative cells	4.5 ± 0.4/3.7–5.8	4.3 ± 0.6/3–5.8
					Heterocysts	6.2 ± 0.2/5.9–7	6.3 ± 0.2/5.8–6.6
					Akinete	8.3 ± 0.7/7–11	9.3 ± 1.3/5.7–12.3
		SBC125	UAM 880	Chott Kralla	Vegetative cells	3.5 ± 0.3/2.8–4.1	5.3 ± 0.6/3.9–6.9
					Heterocysts	5.7 ± 0.4/4.9–6.5	6.5 ± 0.8/4.8–8.9
					Akinete	6.9 ± 0.6/5.4–8	9.8 ± 1.4/6.2–12.6

## Data Availability

Not applicable.

## Supplementary Materials

The following are available online at https://www.mdpi.com/2076-2607/9/3/487/s1, Figure S1: Rarefaction curves showing (a) Observed OTUs, (b) Chao1 and (c) Good’s coverage alpha diversity indices.
