# Supplementary material for: Microenvironmental Conditions Drive the Differential Cyanobacterial Community Composition of Biocrusts from the Sahara Desert
Source: Microorganisms. 2021 Feb 25;9(3):487. doi: 10.3390/microorganisms9030487 (PMC7996595; doi:10.3390/microorganisms9030487)

microorganisms

MDPI

Supplementary Materials: Microenvironmental Conditions Drive the Differential Cyanobacterial Community Composition of Biocrusts from the Sahara Desert

Smail Mehda [1,2,3], M. Ángeles Muñoz-Martín [1], Mabrouka Oustani [4], Baelhadj Hamdi-Aïssa [2], Elvira Perona [1] and Pilar Mateo [1,*]

Figure S1. Rarefaction curves showing (a) Observed OTUs, (b) Chao1 and (c) Good's coverage alpha diversity indices.

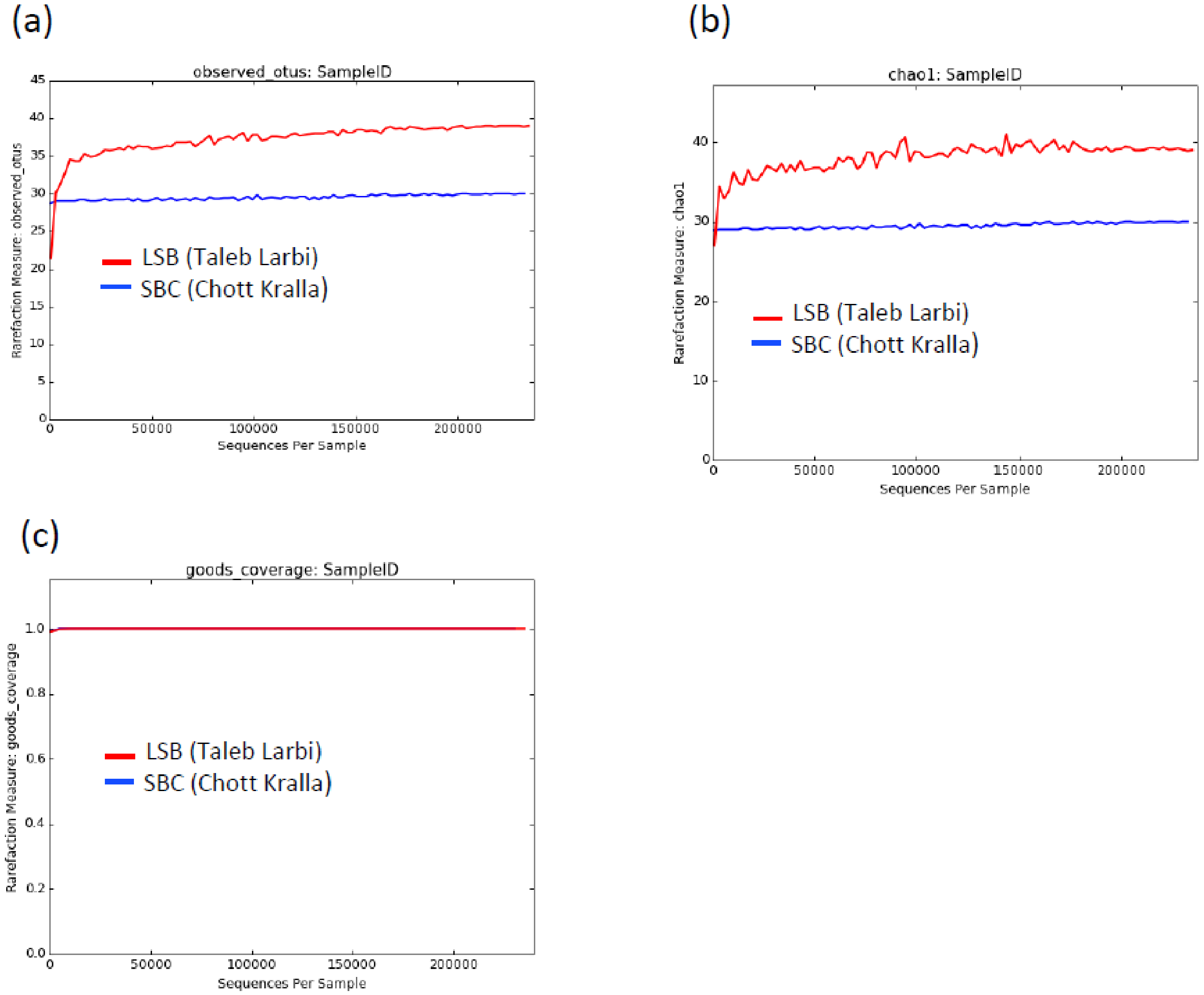

Supplement: Supplementary file 1 [file microorganisms-09-00487-s001.pdf]
